# Stability in change: building a stable ecological security pattern in Northeast China under climate and land use changes

**DOI:** 10.1038/s41598-024-63391-3

**Published:** 2024-06-02

**Authors:** Boyan Zhang, Hui Zou, Detai Duan, Xinyu Zhou, Jianxi Chen, Zhonghua Sun, Xinxin Zhang

**Affiliations:** 1 School of Life Sciences and Technology, Heilongjiang Genuine Wild Medicinal Materials Germplasm Resources Research Center, Harbin Normal University, Harbin, China; 2 Heilongjiang Seed Industry Technology Service Center, Harbin, China

**Keywords:** Ecological security pattern, Global change, Northeast China, Ecosystem services, Ecology, Ecological networks, Ecosystem services

## Abstract

Climate change and land use change caused by human activities have a profound impact on ecological security. Simulating the spatio-temporal changes in ecosystem service value and ecological security patterns under different carbon emission scenarios in the future is of great significance for formulating sustainable development policies. This study quantified the four major ecosystem services (habitat quality, water retention, soil erosion, and carbon storage) in Northeast China (NC), identified ecological source areas, and constructed a stable ecological security pattern. The results show that the spatial patterns of soil erosion, carbon storage, water retention, and habitat quality, the four major ecosystem services in NC, are relatively stable in the next 30 years, and there is no significant difference from the current spatial pattern distribution. The SSP1–2.6 carbon emission scenario is a priority model for the development of NC in the next 30 years. In this carbon emission scenario, the NC has the largest ecological resources (191,177 km^2^) and the least comprehensive resistance value (850.006 × 10^−4^). At the same time, the relative resistance of the corridor in this scenario is the smallest, and the area of the mandatory reserve pinch points is the least. The ecological corridors in the SSP1–2.6 scenario form a network distribution among the ecological sources, connecting several large ecological sources as a whole. This study fills the knowledge gap in building a stable ecological security pattern in NC under the background of global change, and provides a scientific basis for the decision-making of regional ecological security and land resource management.

## Introduction

Ecological security, including the maintenance of ecosystem services and the promotion of human well-being, stands at the nucleus of sustainable human development^1^. The rapid global change spurred by human activities has engendered numerous environmental problems, including the overexploitation of natural resources and the loss of species diversity caused by land use change, alongside extreme climatic occurrences induced by climate change. The synergy of the two has aggravated the degradation of ecosystem services in various regions across the globe. Given that the assessment of ecological security pattern can reveal the phenomenon of blind restoration and inefficient protection in ecological conservation endeavors, while also offering viable pathways to effectively mitigate conflicts between economic progress and ecological preservation, sustain biodiversity stability, and foster regional sustainability^2^. Consequently, the adoption of ecosystem services as a framework for regional ecological security assessments has garnered widespread attention. In the past few decades, many ecological security assessment studies have been carried out in different regions, especially in East Asia, Central Europe and North America^3^. Unfortunately, a recent global meta-analysis shows that there are only a few dozen studies on ecological security under climate and land use change, accounting for only 5% of the total studies^3^. This greatly limits our understanding of the intricate relationship between global change and regional ecological security. Therefore, it is urgent to conduct ecological security assessment studies in more ecologically fragile areas, which is of great significance to the construction of regional ecological civilization^4^.

Ecological security Pattern (ESP), also known as the ecological security network, is one of the bases of regional ecological security assessment^5^. ESP is mainly composed of ecological source, ecological corridor, and ecological pinch points^6^. The identification of ecological source areas, crucial for constructing ESP, is typically categorized into two methods. The first involves directly designating areas such as protected zones, parks, and water bodies as ecological source areas^7,8^. However, this method greatly increases human subjectivity and has certain limitations. The other method employs various ecosystem service assessments to determine ecological source areas^3,9, 10^. Compared with the former, this approach offers greater objectivity and scientific rigor, thus providing a better reflection of actual ecosystem conditions. By utilizing ecological source areas and resistance surfaces, ecological corridors can be established, effectively enhancing habitat patch connectivity^11,12^. Most existing studies construct resistance surfaces by assigning values to different land use types based on expert experience^10,13^. However, this method ignores the spatial differences of the same land use type, and cannot objectively and scientifically describe the obstacles of species migration. The area with higher current value in the ecological corridor is the ecological pinch points, which has a higher probability of passing through in the process of species migration^14^. At present, the most commonly used models for quantitative study of ecological corridors include minimum cumulative resistance (MCR), least cost path (LCP) and circuit theory^15–17^. Among them, MCR model does not specify the key pinch points of the corridor, while the combination of circuit theory and LCP model improves the above deficiencies, increasingly serving as a quantitative research on ecological corridors and ecological pinch points^18^.

Regional scale is the focus of research on ecological security pattern at the spatial level, and relatively complete physical geographic regions are of great significance for the study of sustainable processes of ecosystems^19^. Northeast China (NC) serves as an irreplaceable ecological barrier in China and even Northeast Asia, with a rich variety of land use types within the region, where forests and grasslands occupy a considerable proportion. These landscapes play a crucial role in improving ecological functions such as biodiversity and water retention in the ecological barrier area^19,20^. At the same time, NC is also a sensitive and vulnerable area to global climate change. Since large-scale reclamation in the 1950s, ecologically functional land such as forests, grasslands, and wetlands have been continuously occupied, developed and destroyed, leading to increasing issues such as water scarcity, declining soil organic carbon content, and degradation of ecosystem services^19,21, 22^. Therefore, building a scientific and reasonable ecological security pattern has become an urgent need for maintaining ecological security and sustainable socio-economic development in NC. However, the assessment of ESP in this climate-sensitive area in NC has only considered the current climate and land use^19^, severely lacking in-depth research on ESP in NC under global change. In this context, this study evaluated four typical ecosystem services (carbon storage, water retention, soil erosion and habitat quality) in NC at present and in the next 30 years, and combined each ecological service layer with entropy weight method and circuit theory model to build an ecological security system in NC. The purposes of this study and the anticipated scientific issues addressed by this study are as follows: (1) Temporal and spatial changes of ecosystem services in NC under different carbon emission scenarios under global change. (2) Identification of source areas of NC under global change. (3) Using least cost path (LCP) and circuit theory to construct a stable ecological security pattern for the Northeast region of China over the next 30 years.

## Methods

### Study area

**Figure 1:**
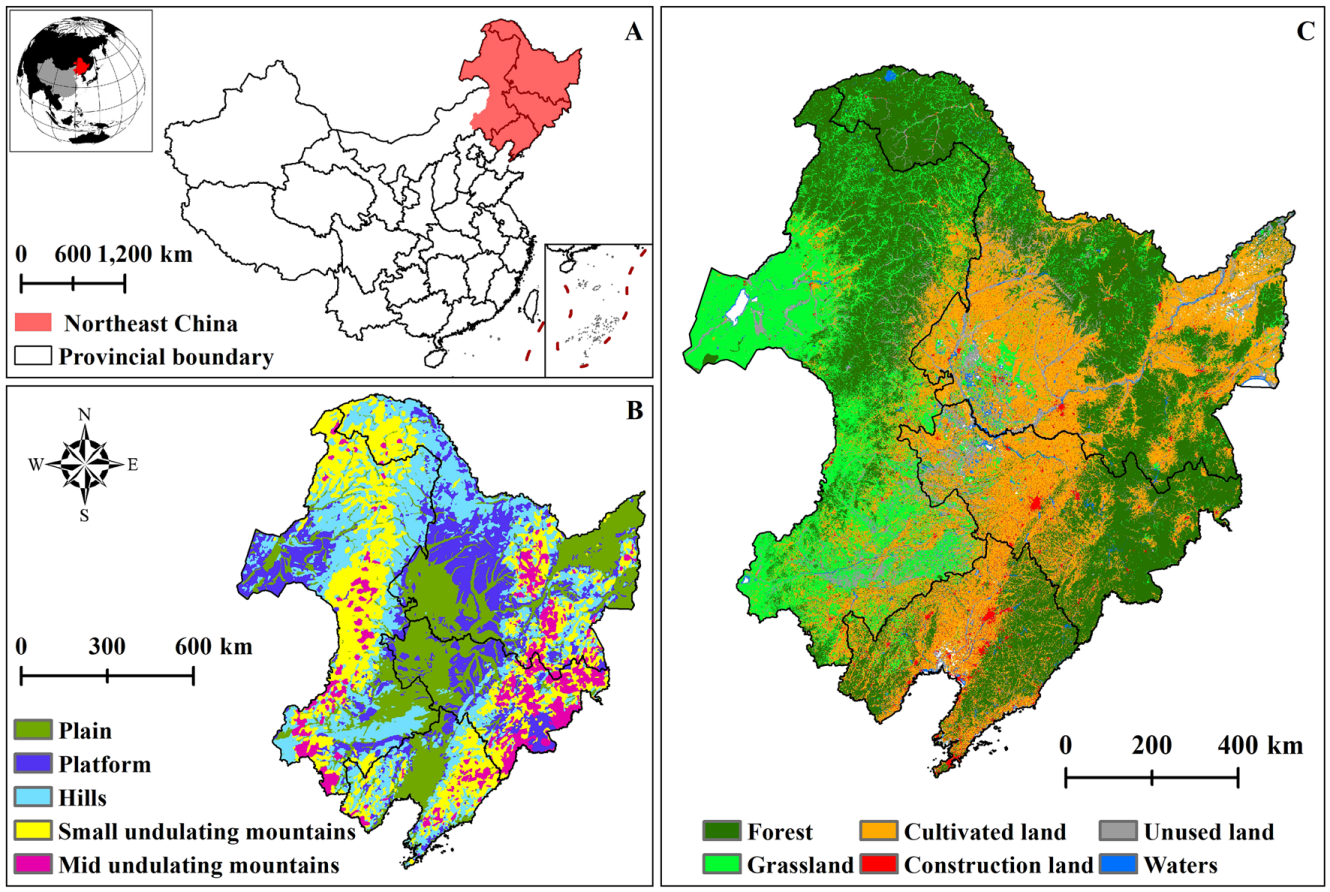
Study area: (**A**) geographical location of NC in China, (**B**) landform of NC, (**C**) land use in NC.

NC is located in the northeast of China (115° 05ʹ–135° 02ʹ E, 38° 40ʹ–53° 34ʹ N), including Heilongjiang Province, Jilin Province, Liaoning Province and the eastern part of Inner Mongolia Autonomous Region. This area spans from the warm temperate zone to the cold temperate zone from south to north and transitions from humid to semi-humid to semi-arid from east to west. The climate type of NC falls under the temperate monsoon climate, characterized by distinct seasons, with warm and rainy summers, and cold and dry winters. It boasts a variety of natural resources and terrain types, including forests, wetlands, grasslands, plains, mountains (Fig. 1). The northwest, northeast and southeast are surrounded by the Greater Khingan Mountains (GKM), Lesser Khingan Mountains (LKM) and Changbai Mountains (CM) respectively, forming a natural boundary around the Northeast Plain.

### Data collection

The current land use data (2020) was downloaded from the Resource and Environment Science and Data Center (https://www.resdc.cn/). The Coupled Model Intercomparison Project (CMIP) aims to better understand past, present, and future climate change^23^. At present, CMIP has entered the sixth stage. It includes the latest generation of integrated earth system model (ESM), which is driven by historical greenhouse gas concentrations and tracks different greenhouse gas and aerosol concentrations in the future according to the shared socio-economic path scenario (SSPs)^24^. There are three typical coupling scenarios in the SSPs of the CMIP6 and they are widely used: Low carbon emission scenario (SSP1–2.6), moderate carbon emission scenario for sustainable development (SSP2–4.5), and high carbon emission scenario for rapid economic development (SSP5–8.5). Therefore, this study selects the future land use data under the scenarios of SSP1–2.6, SSP2–4.5 and SSP5–8.5 in the research results of Hou et al.^25^ (10.6084/m9.figshare.20088368.v1). The land use data used include current and future scenario simulations from different sources. The secondary classification of land use data from different sources is different. In order to unify the land type, we use the primary classification of land use data. This helps to compare different scenarios in subsequent correlation analysis. This classification method is widely used in related research^26–28^.

The altitude data, current (2020–2021) and future (2030s, 2050s) precipitation data were downloaded from the WorldClim (www.worldclim.org). In order to be closer to China’s climate, BCC-CSM2-MR model was selected for the future precipitation data. Current (2020) and future (2030s, 2050s) evapotranspiration data were downloaded from the National Earth System Science Data Center (http://www.geodata.cn/). The soil data was from the Harmonized World Soil Database v 1.2 (HWSD) released by the Food and Agriculture Organization of the United Nations (https://www.fao.org). In order to be more representative of soil properties, data from subsoil (30–100 cm) were used in subsequent calculations^29^. The bedrock depth was downloaded with the 30s resolution released by Yan et al.^30^. Road data was downloaded from the Resource and Environment Science and Data Center. Then we used Euclidean distance to calculate the distance road data. In order to ensure data consistency, all spatial data were resampled to the resolution of 30s.

### Landscape pattern analysis

Habitat fragmentation refers to the process that the entire large-area suitable area of species continuously distributed gradually shrinks and splits into two or more smaller suitable area patches under the interference of human activities or natural environment^31^. Our study used landscape indices to quantify the current and future fragmentation degree of NC. Seven landscape indices were selected, including habitat patch area and quantity index (the number of patches (NP), the patch density (PD), the mean patch size (AREA_MN)), habitat geometry index (the area-weighted mean shape index (SHAPE_AM), the area-weighted mean patch fractal dimension (FRAC_AM)) and landscape dispersion index (the division index (DIVISION), and the aggregation index (AI)). The above indices were calculated by no sample method in Fragstats 4.2 software.

### Ecosystem services assessment

Considering the factors of land use type, climate, soil and topography, we used the revised universal soil loss equation (RUSLE) to analyze the changes of soil erosion caused by global warming and socio-economic development of human activities in NC. Then, in order to explore the current and future changes of carbon storage, water retention, and habitat quality in NC, we used the Annual Water Yield, Carbon Storage and Sequestration, and Habitat Quality Model in InVEST 3.11.0 to quantify them^10^. More details on these calculations can be found in the supplementary document (Table S2).

### Construction of ecological security pattern

This study developed the ecosystem assessment model of NC under the scenarios of SSP1–2.6, SSP2–4.5 and SSP5–8.5 carbon emissions in the next 30 years. The model investigates the ecological connectivity among key habitats in the NC, offering theoretical support for ecological conservation and management in the area.

#### Identification of ecological sources

Ecological sources are key ecological patches that promote ecological processes and provide high-quality ecosystem services^32^. In this study, ArcMap 10.5 was used to normalize the ecosystem service layers, and then the core area zonation algorithm in Zonation 4.0 software (http://cbig.it.helsinki.fi/software/) was used to overlay the ecosystem service layers. Specifically, the weight of each ecosystem service layer was set to 1, and other parameters were the default values. In addition, in order to optimize the obtained results, we set the warping factor to 1, that is, one mesh is removed at a time^33^. Taking the current and future natural landscape areas in NC as the scope, ArcMap 10.5 was used to screen out the top 25% ecosystem service functions as the premise to determine the ecological resources. Due to the small size of ecological patches, which lack connectivity between them, it is challenging to conduct conservation efforts effectively^10^. In this study, we ultimately selected ecological source that met the above criteria and had an area greater than 10 km^2^.

#### Establishment of resistance surface

The resistance surface reflects the level of difficulty for species migration between ecological sources^18^. When constructing the resistance surface, most studies mainly assign resistance values to factors such as land use types, elevation, slope, and roads based on expert knowledge^6,34^. However, this method fails to account for internal differences within the same land use type^10^. In previous studies, the restrictions were mainly divided into natural environmental factors and social–economic factors. Among them, elevation and slope are more widely used as natural environmental factors, and distance from construction land and road are more widely used as social–economic factors^10,19, 28^. On this basis, soil erosion and water retention, based on land use data and climate data, were incorporated as resistance factors. Therefore, six resistance factors were used in this study: soil erosion, slope, distance from construction land, distance from road, water retention capacity, and elevation. In order to eliminate the dimensional influence between each index, we normalized each resistance surface using ArcMap 10.5. We used the entropy weight method to determine the weight of each resistance, which is an objective weighting method and not affected by the evaluator preference^35^. Finally, the grid calculation tool in ArcMap 10.5 was used for weighted superposition to obtain the comprehensive resistance surface. The higher the resistance value, the greater the obstacles or costs of biological migration.

#### Determination of ecological corridors and pinch points

At the beginning of the twenty-first century, circuit theory was used to simulate the gene flow and movement route of organisms^36^. The theory is based on Ohm's Law, which has the following formula^11,36^:

$$I=\frac{V}{{R}_{ef}},$$

where *I* refers to the net probability of gene flow or species movement; *V* represents the probability of leaving a source for a given target source; *R*_*ef*_ reflects the degree of obstruction of species movement or energy flow. In this study, Circuitscape 4.0.5 and Linkage Mapper Toolkit in Arcmap10.5 was used to implement circuit theory. This approach is now widely used to analyze habitat connectivity^37^. The detailed identification process of ecological corridors and ecological pinch points is as follows. First, in the Linkage Mapper Toolkit’s Build Network and Map Linkages module, we inputted the ecological sources and comprehensive resistance surface maps. Following the approach of Han et al.,^38^ we set the threshold to 200 km and left other parameters as default settings, calculating the cost-weighted distance (CWD) and least cost path (LCP) between ecological sources. Both the length of the LCP and CWD are considered to be more appropriate means of connectivity between ecological sources^39^. Then, using Circuitscape 4.0.5 and the Pinchpoint Mapper in the Linkage Mapper Toolkit, we generated a current map to identify pinch points and categorized them into four levels using natural breakpoints, with the highest level of current density designated as ecological pinch points. Some studies have pointed out that the adjacent pair model of the studied area has little value for the overall landscape pattern, as organisms can detour through other core areas and move between the two core areas. Therefore, this study adopts raster centrality to identify the pinch points^40^. Since the corridor width does not affect the location of pinch points and regional connectivity, the weighted cost distance of 1 km was set as the corridor width in this study.

## Result

### Changes in landscape pattern

Landscape pattern index analysis shows that the seven landscape pattern indexes in NC will increase or decrease year by year under different carbon emission scenarios in the future, showing a trend of gradual aggregation. In 2030s and 2050s, NP and PD will decrease significantly compared to the current under the three carbon emission scenarios; under the SSP1–2.6 carbon emission scenario, NP will decrease by 75.496% and 80.948%, respectively, and the PD will decrease by 75.489% and 80.902%; under the SSP5–8.5 carbon emission scenario, NP will decrease by 77.208% and 79.311%, and the PD will decrease by 77.143% and 79.248%, respectively. In terms of the AREA_MN, NC will significantly increase under the three carbon emission scenarios in the future; under the SSP1–2.6 carbon emission scenario, AREA_MN will increase by 308.100% and 424.886% in 2030s and 2050s, respectively; under SSP5–8.5, it will increase by 338.748% and 383.694%, respectively. From the perspective of two geometric shape indexes (SHAPE_AM, and FRAC_AM), the two indexes of NC will decrease compared with the current under three carbon emission scenarios in 2030s and 2050s. Among them, compared with the present, the SHAPE_AM of SSP1–2.6 in 2030s and 2050s will decrease by 41.059% and 45.235%, respectively. and the FRAC_AM will decrease by 1.698% and 1.929% respectively; under SSP5–8.5 in 2030s and 2050s, the SHAPE_AM will decrease by 39.621% and 44.167%, and the FRAC_AM decreased by 1.499% and 1.961%, respectively. From the perspective of DIVISION and AI, which represent landscape dispersion and agglomeration, these two indices in NC will show a trend of aggregation under different climate scenarios in the future; under SSP1–2.6, the DIVISION in 2030s and 2050s will be reduced by 7.439% and 8.610% compared with the current, and the AI will be increased by 27.888% and 30.198%; under SSP5–8.5, the DIVISION in 2030s and 2050s will decrease by 8.827% and 9.206%, and the AI will increase by 28.155% and 29.223% compared with the current (Table S2).

### Temporal and spatial changes of ecosystem services

#### Soil erosion

**Figure 2:**
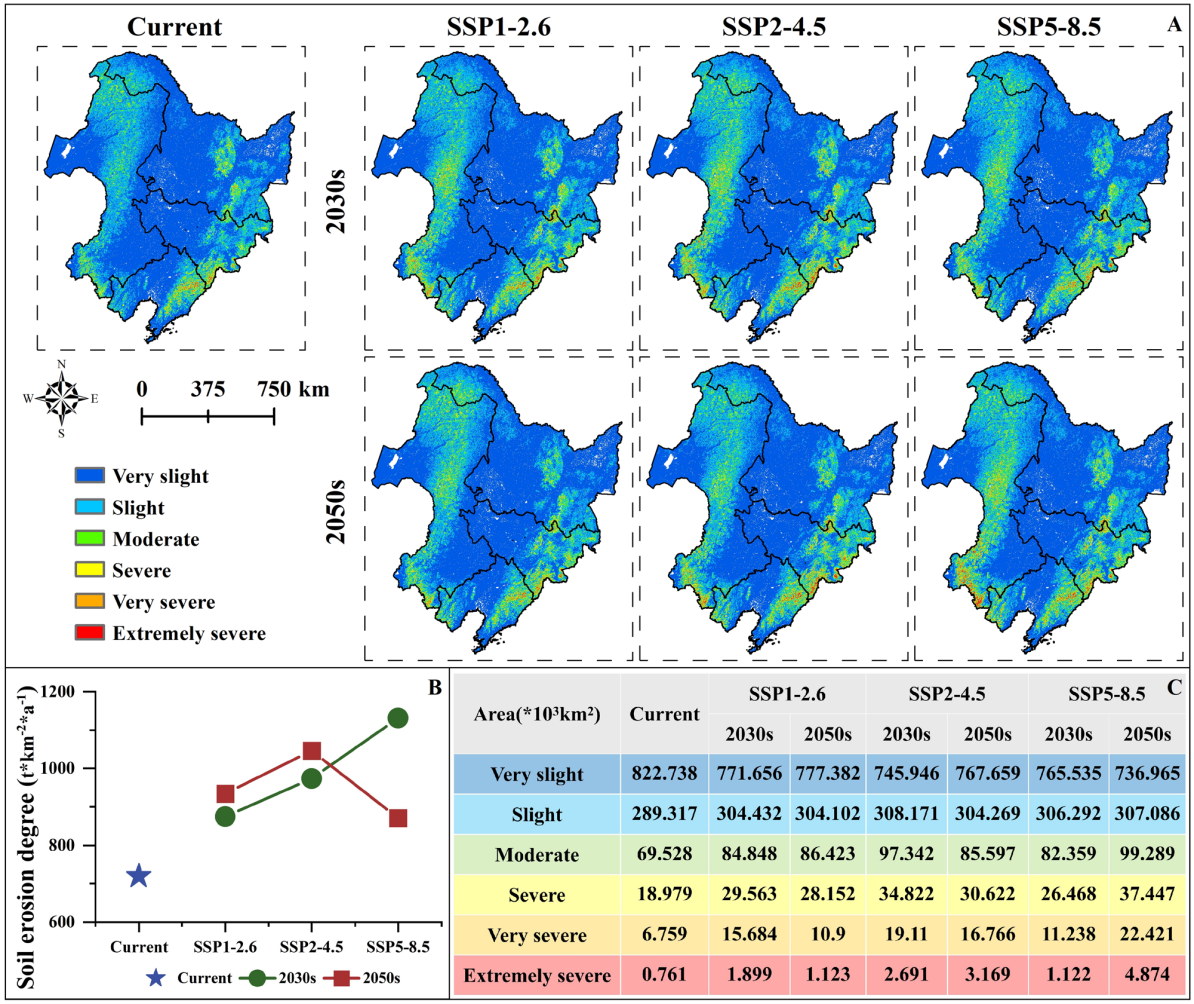
Soil erosion in NC during the current, 2030s and 2050s: (**A**) Spatial distribution of soil erosion in NC during the current, 2030s and 2050s. (**B**) Trends in soil erosion in NC during the current, 2030s and 2050s. (**C**) Changes in soil erosion area at different levels in NC during the current, 2030s and 2050s.

At present, the average soil erosion degree in NC is 719.214 t km^−2^ a^−1^, among which Liaoning Province has the most serious soil erosion degree, which is about 1126.919 t km^−2^ a^−1^. It is followed by Jilin Province (835.738 t km^−2^ a^−1^) and Inner Mongolia Autonomous Region (775.612 t km^−2^ a^−1^). Heilongjiang Province has the weakest soil erosion degree, about 482.781 t km^−2^ a^−1^ (Table S3). In the next 30 years, soil erosion in both the overall and provincial will be worse than the current, with progressively worse expansion areas mainly distributed in eastern Jilin Province, western and eastern Liaoning Province, central and southern Inner Mongolia Autonomous Region (Fig. 2A). Under different climatic scenarios, the province with the most severe soil erosion remains Liaoning Province, while the province with the least soil erosion continues to be Heilongjiang Province. As can be seen from Fig. 2B and Table S3, under the scenario of SSP1–2.6 carbon emissions scenario, the average erosion degree of NC in 2030s and 2050s will increase by about 29.834% and 21.651% compared with the current. The area with low soil erosion will decrease by about 51.082 × 10^3^ km^2^ and 45.356 × 10^3^ km^2^, respectively, while the area with severe soil erosion will increase by about 149.540% and 47.569%, respectively. Under the SSP2–4.5 carbon emission scenario, the average erosion degree in 2030s and 2050s will increase by 45.416% and 35.352%, respectively, compared with the current. And the area of very slight soil erosion degree will change to 745.946 × 10^3^ km^2^ and 767.659 × 10^3^ km^2^, while the region experiencing extremely severe soil erosion will change to 2.691 × 10^3^ km^2^ and 3.169 × 10^3^ km^2^. Under the scenario of high carbon emission of SSP5−8.5, the soil erosion degree in NC gradually intensifies progressively over the years. By 2050s-SSP5–8.5 climate scenario combination, the average erosion degree increases to about 1131.946 t km^−2^ a^−1^. The area with very slight soil erosion decreased to the smallest (736.965 × 10^3^ km^2^), while the area with extremely severe soil erosion expands to its maximum extent (4.874 × 10^3^ km^2^) (Fig. 2C).

#### Habitat quality

**Figure 3:**
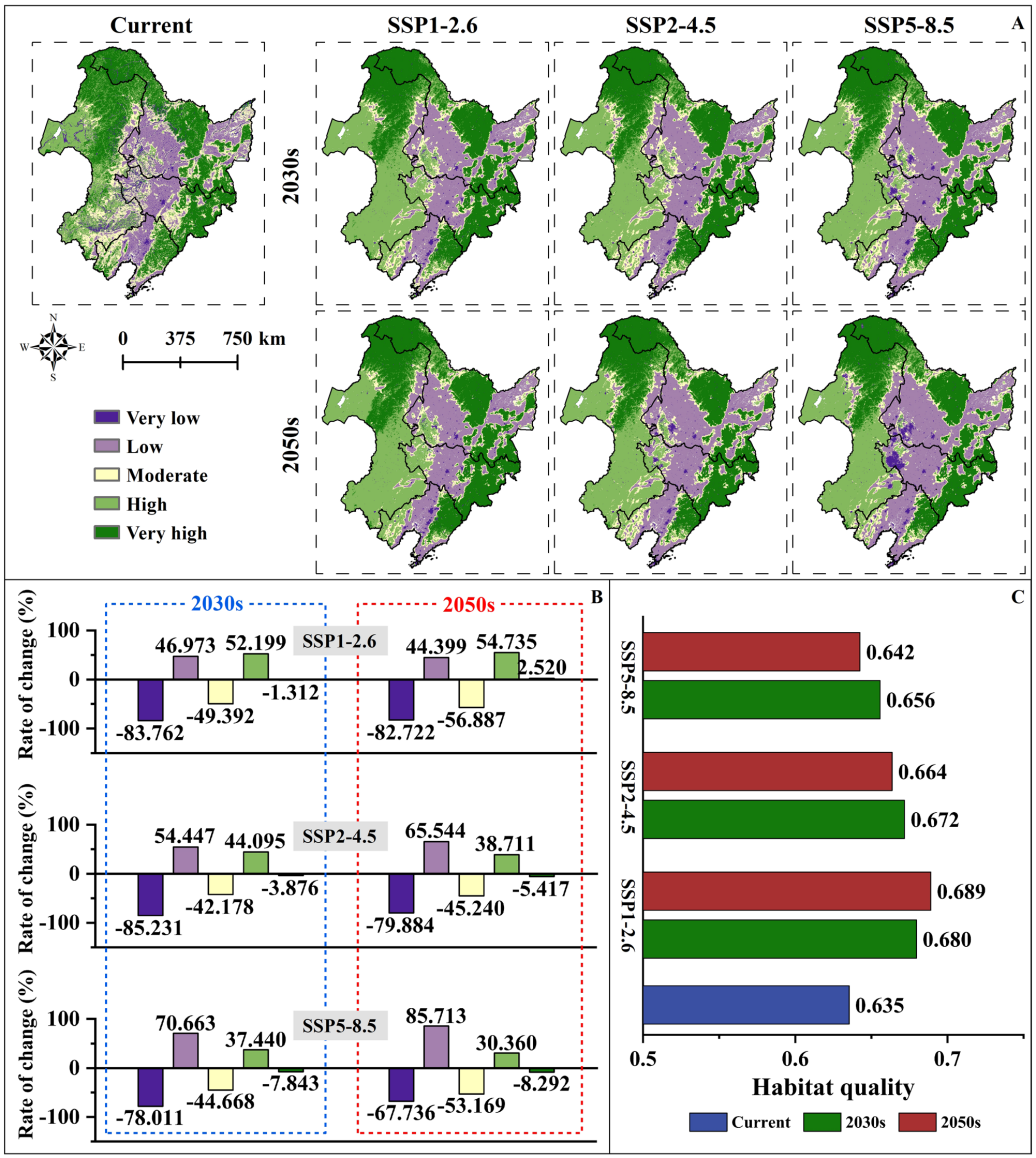
Habitat quality of NC in the current, 2030s and 2050s: (**A**) Spatial distribution of habitat quality in NC in the current, 2030s and 2050s. (**B**) Changes of habitat quality area at different levels in NC in the current, 2030s and 2050s. (**C**) Changes of habitat quality in NC in the current, 2030s and 2050s.

In NC, habitats of very high and high quality are predominantly distributed in forests and grassland. Habitats with moderate quality are primarily situated in the peripheral zones of forests and grasslands. Areas characterized by low and very low quality are mainly located in cultivated and construction land. At present, the overall habitat quality value is about 0.635. In the Inner Mongolia Autonomous Region, the proportion of forest land and grassland is the largest among the highest habitat quality value (0.692); Liaoning Province has the largest proportion of cultivated land and construction land, and the lowest habitat quality value (0.543) (Fig. S1, Fig. 3A, Table S4). Compared with the current situation, the overall habitat quality in NC has improved under various climate scenarios in the future, but in different years, with the increase of carbon emissions, the overall habitat quality values in NC and provinces are projected to be decreased year by year (Fig. 3C). In the 2030s, the habitat quality value of NC is about 0.680 under the scenario of SSP1–2.6 low carbon emissions, and the area of very high quality area is 1.312% less than the current; under SSP2–4.5 and SSP5–8.5, the habitat quality values are about 0.672 and 0.656, and the area of very high quality area is reduced by 3.876% and 7.843%, respectively. In the 2050s, the habitat quality value of NC is about 0.689 under SSP1–2.6, and the area of very high quality area is increased by 2.520% compared with the current; under SSP2–4.5 and SSP5–8.5, the habitat quality values in NC are about 0.664 and 0.643, and the area of very high quality area is reduced by 5.417% and 8.292%, respectively (Fig. 3B).

#### Carbon storage

**Figure 4:**
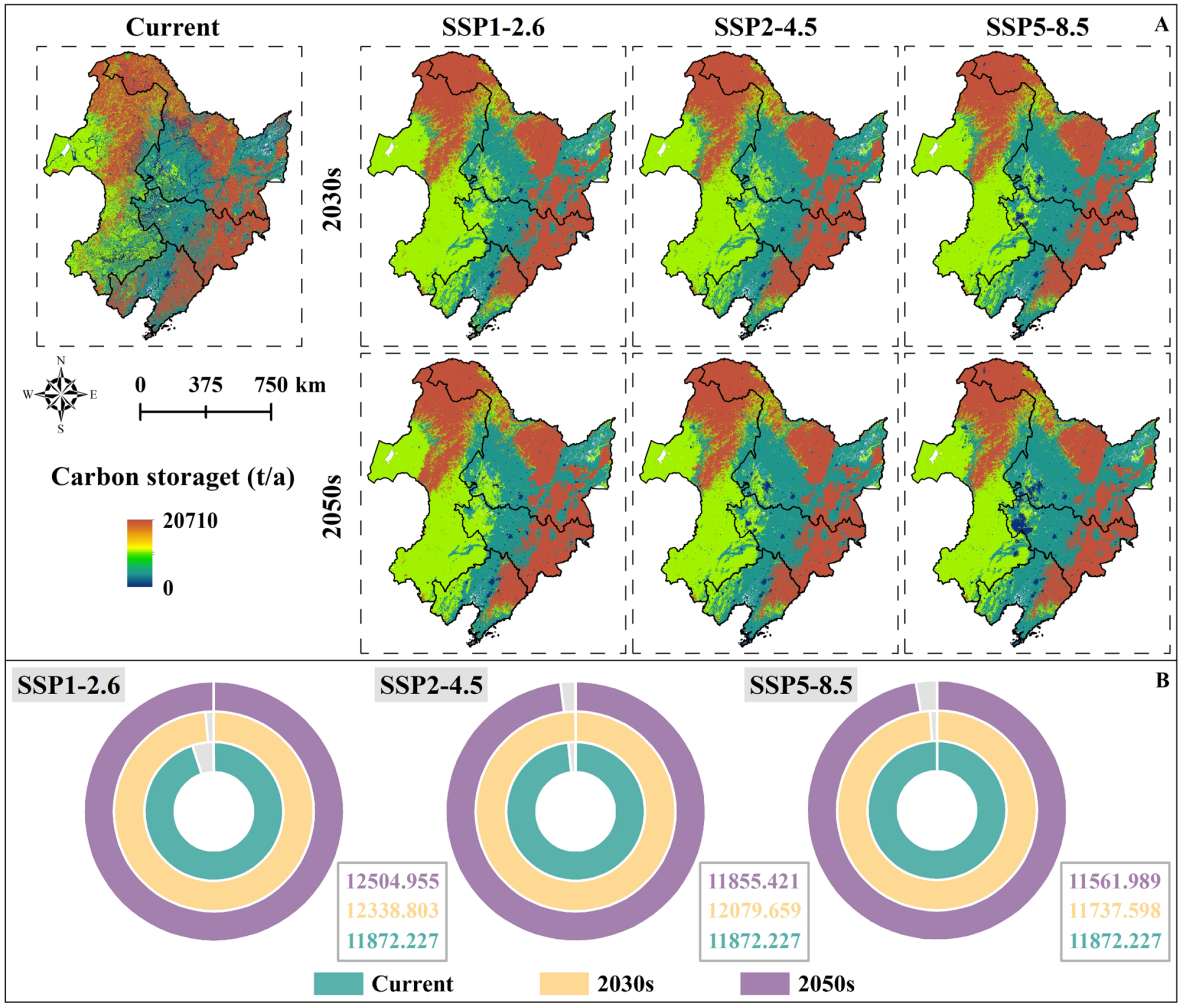
Carbon storage in NC in the current, 2030s and 2050s: (**A**) Spatial distribution of carbon storage in NC in the current, 2030s and 2050s. (**B**) Changes in carbon storage in NC in the current, 2030s and 2050s.

The forest in the north and east of NC has the highest carbon storage, followed by the grassland in the west of NC (Fig. S1, Fig. 4A). Under the current climate scenario, the carbon storage is 12,343.025t/a, and Liaoning Province has the lowest carbon storage 11,180.673 t/a (Table S5). In the future, the carbon storage of NC will have different trends with different carbon emission concentrations. Under SSP1–2.6, the carbon storage of NC will gradually increase with the increase of years, and the carbon storage will increase to 12,504.955 t/a in 2050s. Under SSP2–4.5, the carbon storage in NC will be slightly recovered in 2030s (12,079.659 t/a), but by 2050s, the carbon storage in NC will decrease to 11,855.421 t/a (Fig. 4B). Under SSP5–8.5, the carbon storage in NC decreased year by year and reduced to 11,561.989t/a in 2050s, about 2.613% less than the current. Overall, due to land use change, the total carbon storage of grassland in NC shows a loss trend with the increase of years under different carbon emission scenarios. The total carbon storage of forest shows an increasing trend only under SSP1–2.6, while it shows a slight decreasing trend under SSP2–4.5 and SSP5–8.5.

#### Water retention

**Figure 5:**
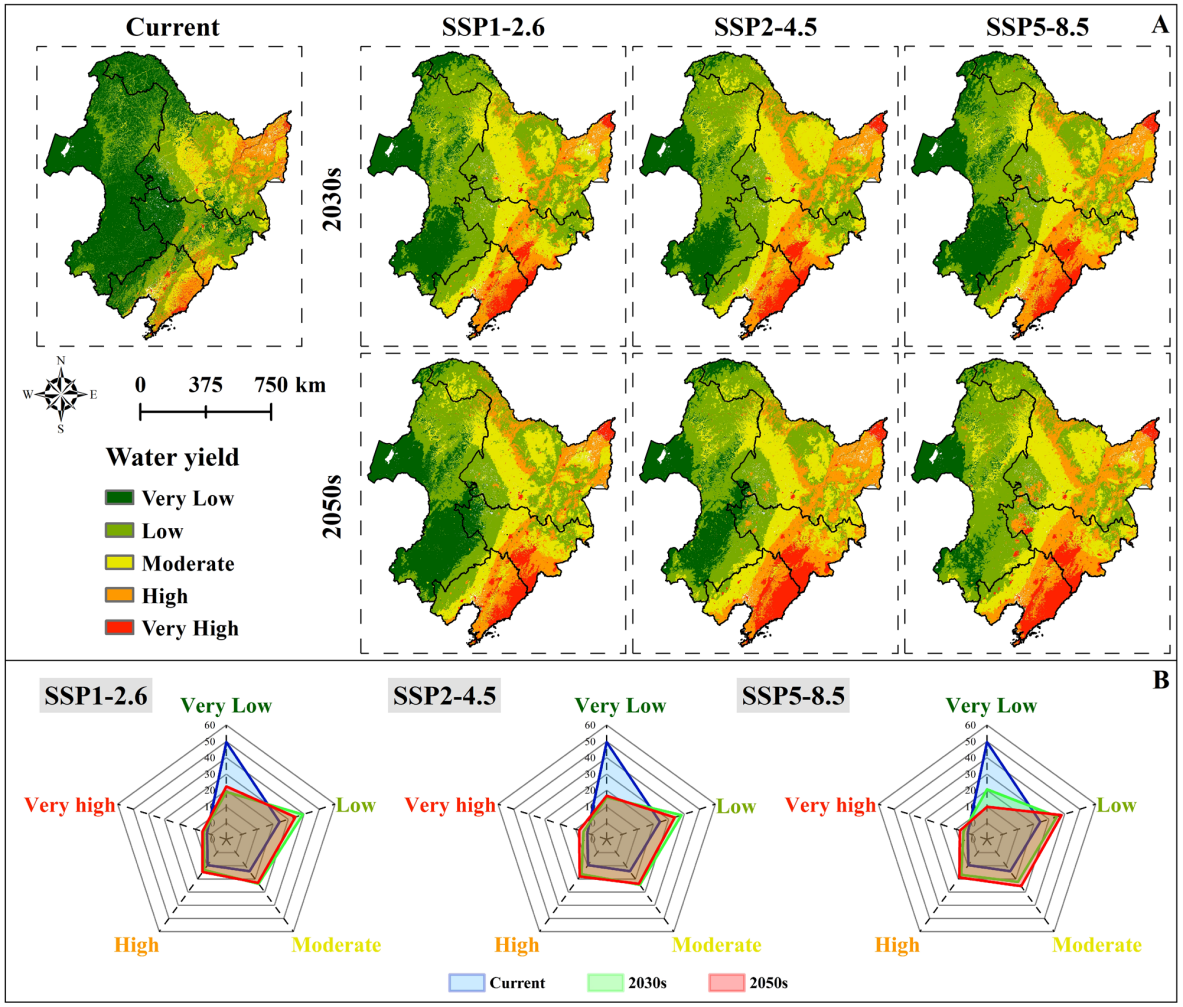
Water retention of NC in the current, 2030s and 2050s: (**A**) Spatial distribution of water retention in NC in the current, 2030s and 2050s, (**B**) Changes in water retention areas at different levels in NC in the current, 2030s and 2050s.

Currently, water retention in NC measures approximately 8.196 mm, with the highest concentration found in the eastern part of Heilongjiang Province and the central and southeastern regions of Liaoning Province, constituting around 2.458% of NC's total area (Fig. 5A). Liaoning Province leads in water retention at 13.376 mm, followed by Heilongjiang Province (11.423 mm) and Jilin Province (8.349 mm), while Inner Mongolia Autonomous Region has the least at 3.306 mm. Projected into the future, water retention in NC is anticipated to increase annually in tandem with rising carbon emissions. The highest water retention, projected at 14.369 mm, occurs in the 2050s under SSP5–8.5, marking a 75.315% increase over the current level. Over the next 30 years, all water retention levels in NC will gradually shift westward, with the very high retention area expanding, particularly in the eastern part of Heilongjiang Province, the southwest and southern part of Jilin Province, and the eastern part of Liaoning Province. Under SSP1–2.6, the very high water retention area is expected to comprise 4.574% and 5.384% of the total area in the 2030s and 2050s, respectively. In SSP2–4.5, the very high water retention area is projected to increase significantly, accounting for 5.062% and 7.689% in the 2030s and 2050s, respectively. In SSP5–8.5, the very high water retention area is estimated to be about 5.211% and 7.518% in the 2030s and 2050s, respectively (Fig. 5B). While water retention in Jilin Province and Liaoning Province is expected to increase over the years under different carbon emission scenarios, Inner Mongolia Autonomous Region and Heilongjiang Province exhibit a decreasing trend under SSP1–2.6 and SSP2–4.5 (Table S6).

### Ecological security pattern changes

**Figure 6:**
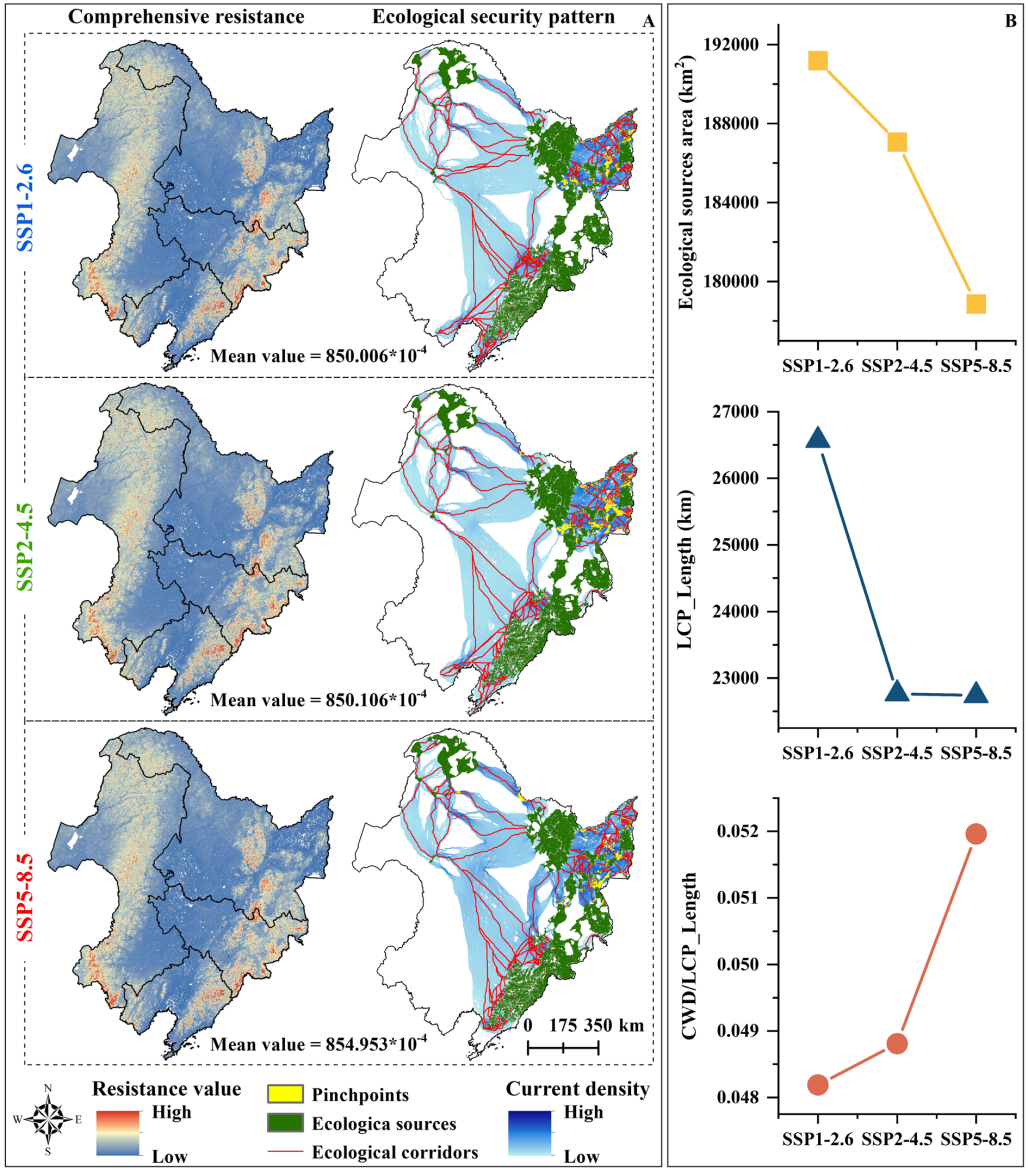
The ecological security pattern of NC under each scenario combination: (**A**) Spatial distribution of the ecological security pattern of NC under each scenario combination, (**B**) Ecological source area, the LCP_Length and the relative resistance change trend of the path movement in NC under each scenario combination.

Over the next three decades, regions in NC exhibiting high comprehensive resistance under various carbon emission scenarios will primarily be situated in the southwest and central areas of the Inner Mongolia Autonomous Region, the central portion of Heilongjiang Province, and the eastern parts of Jilin and Liaoning Provinces (Fig. 6A). The resistance value in NC is expected to rise in correlation with increased carbon emissions, with the smallest comprehensive resistance value observed in the low carbon emission scenario of SSP1–2.6 (approximately 850.006 × 10^−4^). In SSP2–4.5, the value is approximately 850.106 × 10^−4^, and under SSP5–8.5, it increases to 854.953 × 10^−4^. When considering various climate scenarios, provinces with the largest comprehensive resistance values are Inner Mongolia Autonomous Region, followed by Jilin Province, while Liaoning Province has the smallest comprehensive resistance value (Table S7). The minimum comprehensive resistance values for Liaoning Province and Jilin Province under of SSP1–2.6 are approximately 832.318 × 10^−4^ and 873.658 × 10^−4^, respectively. Under SSP2–4.5, the values for other provinces are approximately 976.500 × 10^−4^ and 714.608 × 10^−4^.

The CWD to LCP_Length ratio, indicative of the relative resistance of species movement along a path, serves as a crucial metric in evaluating ecological security. Higher ratios correspond to increased resistance, elevated costs, and reduced channel connectivity. This study reveals that, under varying carbon emission scenarios, the spatial pattern of ecological security in NC is expected to remain relatively stable over the next 30 years. Stable natural terrestrial ecological sources are mainly concentrated in the CM and the LKM, with limited presence in the north of the GKM, indicating high habitat quality and relatively complete ecosystem service functionality. However, the area of ecological sources and the length of ecological corridors are projected to gradually decrease with escalating carbon emissions. Under SSP1–2.6, the area of ecological sources is the largest at about 191,177 km^2^, with an ecological corridor length of approximately 26,570.442 km. Conversely, under SSP5–8.5, the area of ecological sources (178,861 km^2^) and the length of ecological corridors (22,738.979 km) are minimized. As carbon emissions increase, the ratio of CWD to LCP_Length in the ecological corridor progressively rises, from 0.048 in the SSP1–2.6 to 0.052 in the SSP5–8.5, indicating poor regional connectivity (Fig. 6B).

### Temporal and spatial distribution of ecological pinch points

**Figure 7:**
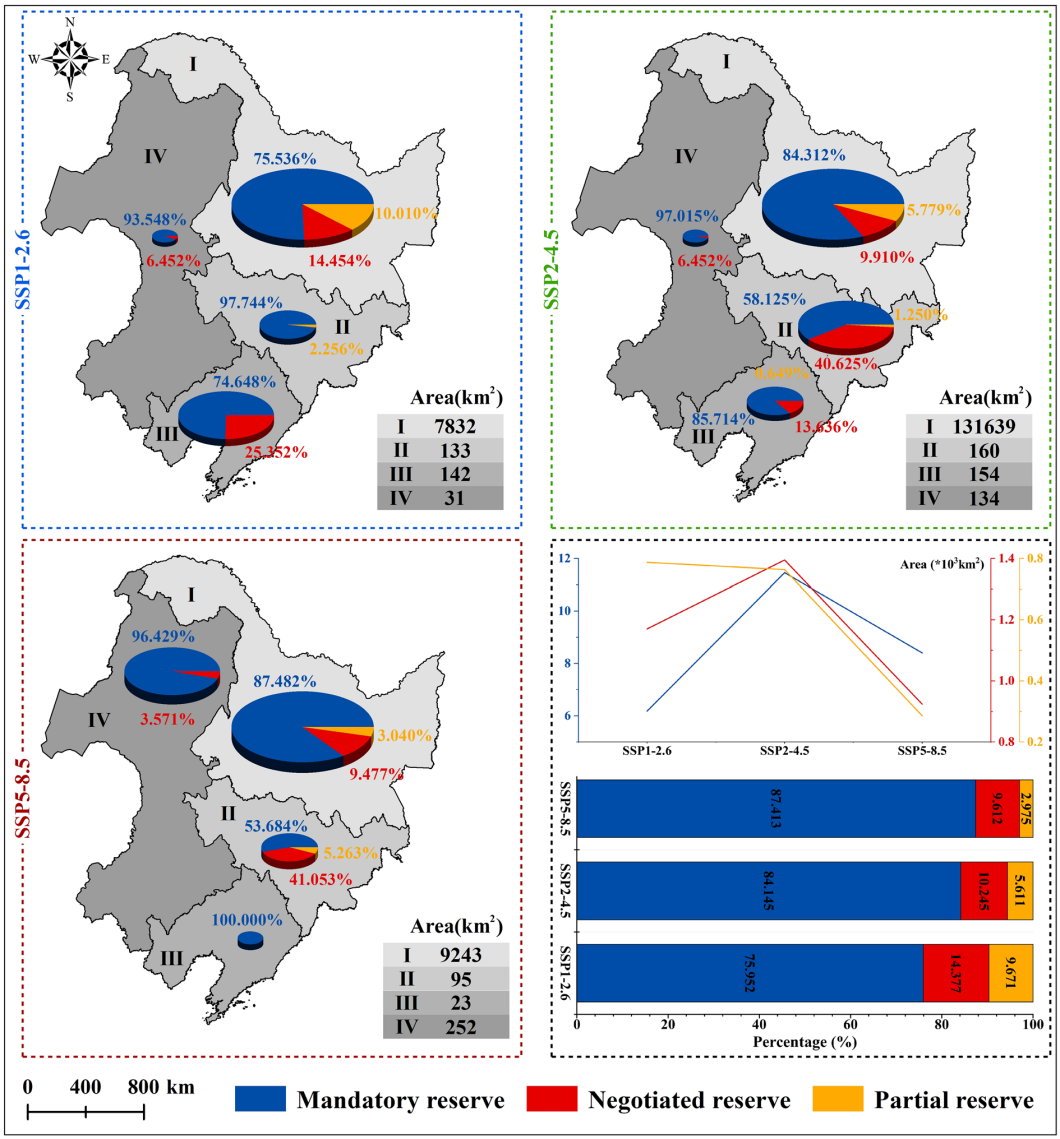
Distribution and change trend of ecological pinch points at different levels under different scenario combinations in NC.

Over the next 30 years, the total area of ecological pinch points in NC exhibits an initial increase followed by a decrease in response to varying carbon emission levels. Under SSP1–2.6, the area measures approximately 8.138 × 10^3^ km^2^, while the SSP2–4.5 sees a 67.326% increase to about 13.617 × 10^3^ km^2^. However, under the SSP5–8.5, the area decreases to about 9.613 × 10^3^ km^2^. Ecological pinch points in NC are predominantly situated in the eastern part of Heilongjiang Province across different carbon emission scenarios, with fewer instances in the southern parts of Jilin and Liaoning Provinces, as well as the eastern part of the Inner Mongolia Autonomous Region (Fig. 6A). With escalating carbon emissions, these pinch points gradually shift northwestward. Under SSP1–2.6, the ranking of pinch point areas in Northeast provinces is as follows: Heilongjiang > Liaoning > Jilin > Inner Mongolia Autonomous Region. However, in SSP5–8.5, the ranking shifts to: Heilongjiang > Inner Mongolia Autonomous Region > Jilin > Liaoning (Fig. 7).

This study categorizes ecological pinch points based on the layers of the ecological service system under different carbon emissions and existing nature reserves in NC. Protection levels are classified as mandatory, negotiated, and partial reserve pinch points, with proportions shifting based on carbon emission levels. In SSP1–2.6, the area of mandatory and negotiated reserve pinch points is 6.181 × 10^3^ km^2^ and 1.170 × 10^3^ km^2^, respectively. Under SSP2–4.5, the area increases to the highest levels at about 11.458 × 10^3^ km^2^ and 1.395 × 10^3^ km^2^, respectively. However, under SSP5–8.5, the area reduces to 8.403 × 10^3^ km^2^ and 0.924 × 10^3^ km^2^, respectively. The proportion of mandatory and negotiated reserve pinch points in the total pinch points increases and decreases with rising carbon emissions. Under SSP5–8.5, the proportions are 87.413% and 9.612%, respectively. Simultaneously, the area of partial reserve pinch points and its proportion in the total pinch points gradually decrease, reaching about 0.286 × 10^3^ km^2^ under SSP5–8.5, accounting for approximately 2.975% of the total pinch points (Fig. 7).

## Discussion

### Method comparison and result uncertainty analysis

In order to better understand the dynamic changes of ecosystem services in NC under global change, the coupling effects of climate and land use change on multiple ecosystem services under three carbon emission scenarios were simulated in this study. Using the LinkageMapperToolkit, we constructed the ecological sources, ecological corridors, and ecological pinch points under each carbon emission scenario in the next 30 years by using the ecological service system under the various climate scenarios. Additionally, we graded the ecological pinch points based on the ecosystem service system layers and existing natural reserves. Compared to existing research (Table 1), this study has some methodological innovations. Compared with the existing research (Table 1), this study has some innovations in methods. First of all, ESP constructed in this study not only considers the current situation but also incorporates global changes, laying the foundation for long-term ecological planning in NC. Second, previous studies on ecological security pattern mainly focused on single factors such as land use or climate change, without fully considering their interactions^41,42^. Third, this study avoids subjective selection of ecological source areas by coupling the current and future four major classical ecosystem services using Zonation, and scientifically selected the area with higher ecological value as the ecological source areas. In addition, the entropy weight method was used to assign the limiting factor when constructing the resistance surface, which makes the resistance surface more scientific and reasonable. Finally, this study chooses to use the combination of circuit theory and LCP model to build an ecological security pattern. Compared with the widely used MCR model, this method can compensate for the lack of MCR in identifying key nodes based on the stochastic movement of organisms^10^, and seek to maximize ecological benefits with minimal cost expenditure. Based on the above methods, we constructed an overall ecological security framework with the whole NC as the study area, and obtain multiple ecological sources belonging to different provinces. Without compromising the integrity of ESP, administrative regions can refer to our results to refine its ESP according to regional natural environment characteristics and social–economic development level, thereby promoting the harmonization of economic and ecological benefits and advancing ecological civilization construction.

**Table 1:** Comparison with previous studies on ecological security.

No.	Time	Study area	Lightspot	Cite
1	Current	Northeast China	Ecological source areas were divided based on ecological services, and ecological security pattern was planned by MCR model	^19^
2	Current	Hexi corridor in China	Plan the ecological security pattern by MCR model	^43^
3	By the 2030s	Haikou, China	Construct the future wetland ecological security pattern based on land use data of different scenarios in the future	^26^
4	By the 2030s	Pearl river delta in China	Use future land use scenarios to identify ecological sources and construct resistance surfaces to build future ecological security early warning	^27^
5	2015–2035	Tibetan plateau of China	Assessment of ecological security pattern based on land use change	^44^
6	By the 2050s	China’s “two ecosystems, three zones”	To explore the impact of future climate change scenarios on ecological protected areas	^28^
7	By the 2050s	Northeast China	Using circuit theory and LCP to construct ecological security pattern of NC under global change in the future	This study

Although this study has contributed to the construction of ecological security pattern, its limitations must be acknowledged. First, the current incompleteness of soil and topographic data may lead to inaccuracy in assessing the status and trend changes of ecosystems, such as temporal discontinuities in soil data and the absence of suitable future modeling results. Second, the absence of data on ecosystem characteristics such as species richness and forest structure has led to a preference for comprehensive assessments based on ecosystem functions and services, which may affect the comprehensiveness of decisions based on the assessment results. Finally, studies at a large geographic scales may overlook detailed local information, while focusing on a small spatial scales may lead to fragmentation effects^45^. However, the most appropriate scale selection has not yet been identified. In light of these considerations, this study chose a relatively complete and species-appropriate physical geographic region (NC) for modeling, but there may still be some limitations. Exploring the internal and external connections at multiple spatial scales is a direction for future research.

### Causes of spatial change of ecosystem services

Consistent with the results of Wang et al.^46^, this study shows that the areas with high carbon storage in NC are mainly distributed in the GKM, LKM, CM, and other forest. This result aligns with the view that forest ecosystems have a high carbon sequestration capacity^46^. Interestingly, our results show that the carbon storage in NC is projected to decline only under the high carbon emission scenario of SSP5–8.5 by 2030ss, which is contrary to the previous studies that China’s carbon storage faces a risk of decline in 2030^47^. This discrepancy may be due to the fact that NC is the largest concentrated distribution area of natural forests in China, serving as a crucial ecological barrier for China and even Northeast Asia. The natural resources of NC’s forests have been attached great importance by the Chinese government, and the protection project of natural forest resources has played an effective role^48^. Under the influence of historical land use change, the loss of carbon storage in Chinese ecosystems has shifted from forest ecosystem to grassland ecosystem^49^. Our results show that the future trend of carbon storage loss in NC is consistent with that in the past, primarily occurring in grassland ecosystems.

The habitat quality in NC presents a distribution pattern with lower habitat quality in the central region and higher habitat quality in the marginal region, which is basically consistent with the results of previous studies^20^. In the future, the degradation areas of habitat quality in NC are mainly distributed around cities^20^, which may be related to frequent human activities and urban expansion. Landscape pattern changes are significant factors influencing habitat quality variations. That is, areas with high landscape connectivity, and widespread distribution of key patches are indicative of overall good habitat quality, facilitating the maintenance of landscape ecological functions^50,51^. The results of this study indicate that the future landscape structure in Northeast China will be significantly more aggregated compared to the current state. This aggregation may be one of the main reasons for the anticipated higher habitat quality in the future compared to the present condition in NC.

This study simulated soil erosion in NC based on the future precipitation and land use data, under the assumption of constant terrain factors. The results were found to be largely consistent with validated data from Wu et al.^52^. At present, the spatial pattern of soil erosion in NC is characterized by higher erosion in forest vegetation area (rugged terrain) and lower erosion in grassland vegetation area (flat terrain), which may be mainly related to slope^46^. Larger slopes increase erosion by intensifying runoff erosion^53^. Some studies have shown that soil erosion intensity in NC will increase in the future^54^, which is consistent with the results of this study. Considering the characteristics of the natural environment in NC, we should pay more attention to the soil erosion in forest in the future. Due to its complex vertical structure, forest vegetation has a strong ability of soil and water conservation^55,56^. However, there is evidence that with the worsening of global climate change, the temperate broad-leaves forest in NC has a tendency to gradually migrate to the boreal coniferous forest in Northwest China^57–59^. During this process, forest vegetation in some areas may experience reduced overall vegetation cover due to unsuitable climate and species migration. Moreover, precipitation in NC is expected to increase in the future, which may aggravate soil erosion in NC. Therefore, in addition to focusing on the protection of existing forests in NC, reasonable expansion of plantation area may as a means to deal with this adverse trend.

Research has shown that soil water retention capacity, a core aspect of ecosystem water conservation ability^60^, is primarily concentrated in the eastern and southeastern parts of Northeast China. Our results are basically consistent with them^46^. Water retention in NC will increase in the future, and areas with higher water retention will gradually expand to the northwest, which is consistent with the future spatial change of precipitation in NC^61^. With the exception of Inner Mongolia Autonomous Region under low carbon emission scenario and Heilongjiang Province under moderate carbon emission scenario, soil water retention capacity across provinces in NC increases over time under different carbon emission concentrations. In NC, the areas with high soil water retention capacity in the future are mainly located in the southeast coastal area with abundant precipitation, and a few are distributed in the inland northeastern Sanjiang Plain area. This region is characterized by concentrated distribution of marsh wetlands in China, and the large area of tower heads and the extremely high content of soil organic matter are one of the reasons for the high soil water retention capacity of soil^62–66^.

In general, the spatial pattern of soil erosion, carbon storage, soil water retention capacity, and habitat quality of the four ecosystem services in NC in the next 30 years is relatively stable, and there is no significant difference from the current spatial pattern. Among them, the spatial pattern of carbon storage and habitat quality was significantly correlated with land use cover type. Forest were the highest carbon storage and habitats quality, followed by grassland, cultivated land, and construction land. In this study, it is predicted that under different carbon emission scenarios, the ecological source areas in NC are mainly distributed in forest, which is highly consistent with the ecological barrier construction of Northeast forest belt proposed by China in the 14th Five-Year Plan and the 2035 Vision Goal. This strong correspondence provides a solid foundation for incorporating ecological security into the management and decision-making process of regional planning, further validates the rationality of the construction of ecological security pattern, and emphasizes the importance of implementing ecological protection measures and policies in future development.

### Impacts of changes in ecosystem services and recommendations for regional future planning

In the next 30 years, NC will have the largest area of ecological resources under the SSP1–2.6 carbon emission scenario, mainly concentrated in forest and grassland. At the same time, under this carbon emission scenario, the ecological corridor .form a network-like distribution between various ecological source areas, connecting major ecological source areas such as the Greater Khingan Mountains, Lesser Khingan Mountains, Changbai Mountains, Sanjiang Plain, and Northeast Plain into a cohesive whole, effectively ensuring the ecological security of the Northeast region. Therefore, the SSP1–2.6 carbon emission scenario should be prioritized in the development of the NC. Due to differences in time and space, there are many uncertainties in the future regional development of ecological protection and restoration measures, so it is particularly important to identify the possible negative impacts of changes in ecosystem services. In this study, the negative effects of global change on ecosystem services in NC are mainly reflected in the decrease of carbon storage in grassland ecosystem and the increase of soil erosion. The decline of grassland carbon storage is often caused by the decline of biodiversity and ecosystem function, and further aggravates the decline and degradation of productivity, forming a vicious cycle, affecting the development of regional animal husbandry^67^. As an important livestock production area in China^68^, the decline of grassland carbon storage will undoubtedly have an economic impact on the livestock dependent cities in the region. Serious soil erosion will seriously threaten regional food production, and soil and water conservation^54^. For example, the loss of black soil caused by soil erosion in Jilin province led to a halving of crop production in the region^69,70^. Unfortunately, the results of this study show that global change may further aggravate this phenomenon. In view of this, we propose the following suggestions, which are expected to provide reference for the maintenance and restoration of the ecology in NC under the background of global change.

#### Protection and restoration of forests and grasslands to enhance regional carbon storage and reduce soil erosion

In forest management, afforestation and the reduction of deforestation are the most significant contributions to the mitigation of climate change^71^. However, in addition to mitigation strategies, adaptation measures to enhance the anti-interference capacity and resilience of forest ecosystems should also be considered^71,72^. Tree species richness is positively correlated with forest productivity and biodiversity^73^. However, forest managers usually simplify forest structure in the process of afforestation. Therefore, we suggest that the composition of the new forest (species and source) should be considered in many aspects when afforesting. In grassland management, the development of animal husbandry and grassland protection is an important contradiction. In recent years, Fang et al.^74^ proposed a new concept of “grass husbandry” with the theory of “preserving the big with the small” as the core, which provides a new perspective for the protection and restoration of degraded grassland in China. According to the content of the concept, it is suggested that high-quality forage varieties should be screened according to regional vegetation characteristics, and experimental artificial grassland should be established initially to reduce the influence of grazing on regional grassland. This is the basis for fundamentally resolving the contradiction between grass and livestock.

#### Increase the area of various types of natural habitats to maintain and improve the quality of regional habitats

Landscape aggregation reduces the contiguous area of land use types, increases landscape connectivity, and improves habitat quality. Patch fragmentation is the most important factor affecting landscape connectivity^51^. Therefore, it is suggested to give priority to restoring grassland area in Northeast Plain (serious fragmentation of natural ecosystem landscape in NC) to reduce landscape fragmentation. In addition, when improving landscape connectivity in NC in the future, besides protecting important habitat patches and increasing the area of natural land types, ecological corridors should also be built to increase the connectivity between the structure and function of habitat patches, reduce the ecological effects brought by fragmentation, better maintain the landscape ecological functions in NC and improve the quality of habitats.

#### Develop strict conservation policies to enhance regional soil water retention capacity

In NC, the Sanjiang Plain exists in the higher soil water retention capacity area, which is the largest area of marsh wetland in China. Wetland protection, as an important part of ecological civilization construction, must be given top priority. We suggest that in the future management of the Sanjiang Plain wetland, human activities should be reduced to prevent soil compaction caused by trampling and vehicle compaction and reduce soil porosity. At the same time, it is suggested to moderately reduce the harvesting of reed in the swamp grass pond, so that more organic residues can enter the ecosystem and form soil organic matter, so as to improve the water retention.

#### Improve the monitoring and protection of ecological safety networks

The width of the corridor has a significant impact on its ecological function. Ensuring the integrity of the ecological corridor system is fundamental to achieving ecological connectivity. It is suggested to take protective measures for the ecological corridor, and make use of the existing natural ecological patches as much as possible to improve the construction efficiency. Strengthen control in the area of mandatory reserve pinch points to prevent damage by human activities. At the same time, environmental monitoring stations are set up nearby to pay timely attention to biological information exchange, animal migration, and changes in the ecosystem. This will help us to promptly detect and respond to any potential ecological issues, promote information sharing, and foster collaboration.

## Conclusion

This study uses an optimization framework to construct a stable ecological security pattern in NC in the context of global change. The results show that under different carbon emission scenarios, the NC performs best under SSP1–2.6 scenario, with the largest ecological resource area, the smallest resistance of ecological corridor, and the smallest area of the mandatory reserve pinch points. In addition, the ecological corridor in this scenario connects important ecological sources in NC, including the GKM, LKM, CM, Sanjiang Plain and Northeast Plain, providing a strong guarantee for the ecological security of the region. This framework can be used as a reference for ecosystem management and protection in other regions. In the future, the impact of biological factors should be further considered, and efforts should be made to optimize and propose a framework that can be used as a reference for ecosystem management and protection in other regions, thereby promoting more scientific, comprehensive, and sustainable regional ecological security planning.

### Supplementary Information

Supplementary Information 1.

Supplementary Information 2.

## Data Availability

The datasets used and/or analyzed during the present study are available from the corresponding author upon reasonable request.
