# Supplementary material for: Stability in change: building a stable ecological security pattern in Northeast China under climate and land use changes
Source: Sci Rep. 2024 Jun 2;14:12642. doi: 10.1038/s41598-024-63391-3 (PMC11144710; doi:10.1038/s41598-024-63391-3)
Supplement: Supplementary file 1 — Supplementary Information 1. [file 41598_2024_63391_MOESM1_ESM.docx]

**Supplementary research methods**

**1 Analysis Content**

(1) Soil erosion;

(2) Habitat quality;

(3) Carbon storage;

(4) Soil water retention capacity;

(5) Identification of ecological source areas;

(6) Construction of comprehensive resistance surface;

(7) Construction of ecological security pattern.


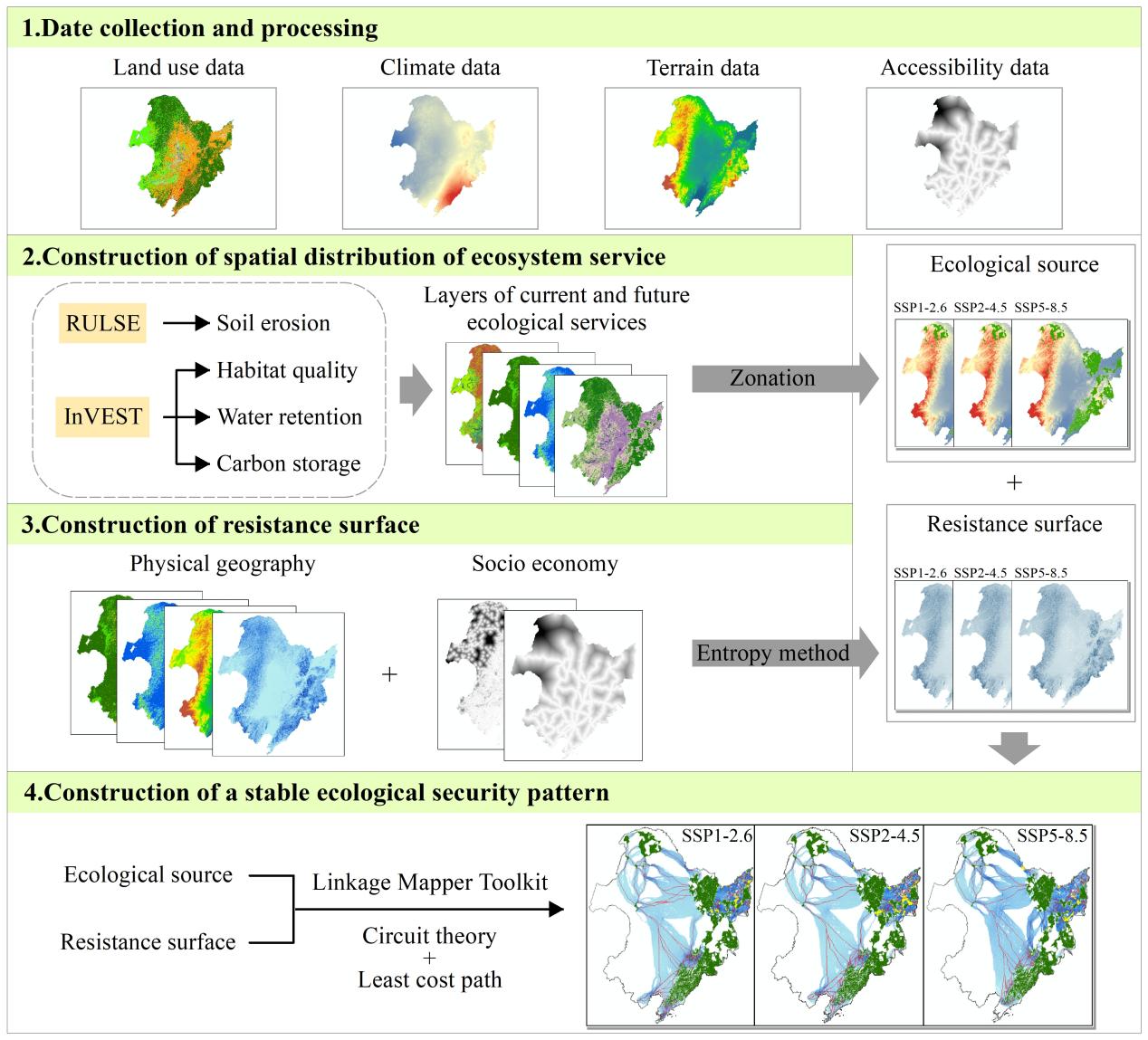


Figure 1: Overall framework of ecological security pattern analysis

**2 Data preparation**

**2.1 Data required for soil erosion modeling**

(1)Current land use (https://www.resdc.cn/) ;

(2)Future land use (https://doi.org/10.6084/m9.figshare.20088368.v1.);

(3)Monthly average precipitation factor (www.worldclim.org);

(4)Elevation data (www.worldclim.org);

(5)Soil data (https://www.fao.org).

**2.2 Data required for habitat quality modeling**

(1)Current land use (https://www.resdc.cn/);

(2)Future land use (https://doi.org/10.6084/m9.figshare.20088368.v1.);

(3)Habitat threat source table, threat source sensitivity table.

**Table 2.2.1 The weight and the maximum influence distance of the threat source**

| THREAT | Maximum influence distance | Weight |
| --- | --- | --- |
| Cultivated land | 8 | 0.7 |
| Construction land | 5 | 0.6 |

**Table 2.2.2 Habitat suitability of different land use types and their sensitivity to various threat sources**

| Land use | Habitat suitability | Sensitivity | |
| --- | --- | --- | --- |
|  |  | Cultivated land | Construction land |
| Forest | 1 | 0.75 | 0.875 |
| Grassland | 0.7 | 0.47 | 0.63 |
| Cultivated land | 0.5 | 0.3 | 0.5 |
| Construction land | 0 | 0 | 0 |
| Unused land | 0.1 | 0.08 | 0.08 |
| Waters | 0.84 | 0.7 | 0.88 |

**2.3 Data required for carbon storage modeling**

(1)Current land use (https://www.resdc.cn/);

(2)Future land use (https://doi.org/10.6084/m9.figshare.20088368.v1.);

(3)Carbon storage table.

**Table 2.3.1 Carbon density values of four major carbon pools**

| Land use | C_above | C_below | C_soil | C_dead |
| --- | --- | --- | --- | --- |
| Forest | 57.4 | 28.8 | 120.9 | 0 |
| Grassland | 30.7 | 24.6 | 54.6 | 0 |
| Cultivated land | 5.9 | 0 | 35 | 0 |
| Construction land | 0 | 0 | 0 | 0 |
| Unused land | 0 | 0 | 0 | 0 |
| Waters | 3 | 3 | 83.7 | 0 |

**2.4 Data required for** **soil water retention capacity**

(1)Annual average precipitation (www.worldclim.org);

(2)Potential evapotranspiration (http://www.geodata.cn/);

(3)Plant root depth (http://globalchange.bnu.edu.cn/research/cdtb.jsp);

(4)Plant available moisture content (https://www.fao.org);

(5)Biophysical table.

**Table 2.4.1 Biophysical table used for the InVEST water yield model**

| Land use | root_depth (mm) | Kc | LULC_veg |
| --- | --- | --- | --- |
| Forest | 3000 | 1 | 1 |
| Grassland | 500 | 0.65 | 1 |
| Cultivated land | 400 | 0.65 | 1 |
| Construction land | 1 | 0.3 | 0 |
| Unused land | 1 | 0.5 | 0 |
| Waters | 1 | 1 | 0 |

**3 Software sources and versions**

(1)InVEST V3.11.0 (<https://naturalcapitalproject.stanford.edu/software/invest>)

(2)ArcMap10.5 (https://www.arcgis.com/index.html)

(3)Zonation 4.0 (http://cbig.it.helsinki.fi/software/)

(4)Circuitscape 4.0.5 (https://circuitscape.org/downloads/)

(5)Linkage Mapper Toolkit (https://linkagemapper.org/)

**4 Operation process**

**4.1 Soil erosion**

Using the grid calculator in ArcMap10.5, calculate land use, monthly precipitation factors, elevation data, and soil data to obtain terrain factors, precipitation erosion factors, plant management factors, and soil erosion factors. Based on the soil erosion equation RUSLE, terrain factors, precipitation erosion factors, plant management factors, and soil erosion factors were calculated using the grid calculator in ArcMap10.5 software to obtain the soil erosion layer and perform analysis and mapping.

**4.2 Habitat quality**

Using the Habitat Quality Model in the InVEST model, input land use data, habitat threat sources, and threat source sensitivity tables to obtain a habitat quality layer. Then, ArcMap 10.5 was used for analysis and mapping.

**4.3 Carbon storage**

Using Carbon Storage and Sequencing in the InVEST model, input land use data, and carbon storage tables to obtain a carbon storage layer. Subsequently, analysis and mapping were conducted using ArcMap 10.5.

**4.4 Water retention**

Using the Annual Water Yield in the InVEST model, input annual precipitation, potential evapotranspiration, plant root depth, available plant water content, and biological table to obtain the soil water retention capacity layer. Subsequently, analysis and mapping were conducted using ArcMap 10.5.

**4.5 Identification of ecological source areas**

Overlay the four classic ecological service systems using the core area zonation algorithm in Zonation 4.0. In this study, the weight of each ecosystem service layer is set to 1, the warp factor is set to 1, and other parameters are the default values of the model. Using ArcMap10.5 to screen out the top 25% of ecological functions, and the area is more than 10 km^2^.

**4.6 Construction of comprehensive resistance**

Soil erosion, slope, distance from construction land, distance from road, soil water retention capacity, and altitude were normalized. Then the entropy weight method was used to determine the weight of each resistance, and the grid calculation tool in ArcMap10.5 was used to weight each element and get a comprehensive resistance.

**4.7 Construction of ecological security pattern**

First, the ecological source and the comprehensive resistance surface map was input into Build Network and Map Linkages in the Linkage Mapper Toolkit, set the threshold to 200 km with other parameters was default. The cost-weighted distance (CWD) and minimum cost path (LCP) between ecological sources are calculated. Then, Circuitscape 4.0.5 and Pinchpoint Mapper in Linkage Mapper Toolkit were used to generate the current diagram for identifying pinch points, and natural break points were used to divide them into four levels. The highest level of current density was ecological pinch points. In this study, raster centrality model was selected and 1 km weighted cost distance was set as the corridor width.
