# Supplementary material for: Stability in change: building a stable ecological security pattern in Northeast China under climate and land use changes
Source: Sci Rep. 2024 Jun 2;14:12642. doi: 10.1038/s41598-024-63391-3 (PMC11144710; doi:10.1038/s41598-024-63391-3)
Supplement: Supplementary file 2 — Supplementary Information 2. [file 41598_2024_63391_MOESM2_ESM.docx]

Table S1 Reference table for abbreviations in the manuscript

| Abbreviation | Full text |
| --- | --- |
| NC | Northeast China |
| LCP | least cost path |
| MCR | minimum cumulative resistance |
| GKM | Greater Khingan Mountains |
| LKM | Lesser Khingan Mountains |
| CM | Changbai Mountains |
| RUSLE | Revised Universal Soil Loss equation |
| CWD | cost-weighted distance |
| ESP | Ecological security Pattern |

Table S2 Ecological Service Formula

| **Ecosystem Service Functions** | **Method** | **Formula and Variable Description** |
| --- | --- | --- |
| Habitat Quality | InVEST habitat quality model | $Q_{xj}=H_{j}\left( 1-\frac{D_{xj}^{2}}{D_{xj}^{2}+k^{2}} \right)$  *Q_xj_* is the habitat quality of grid *x* in land use type *j*; *H_j_* represents the habitat suitability of land use and land cover *j*; *k* is a semi saturated parameter; When$1-\frac{D_{xj}^{2}}{D_{xj}^{2}+k^{2}}$ = 0.5, the value of *k* is equal to the value of *D*. |
| Water Yield | InVEST Annual Water Yield model | $Y_{xj}=P_{x}\left( 1-\frac{AET_{xj}}{P_{x}} \right)$  *Y_xj_*: water yield of raster *x* in land use type *j*; *Px*: annualaverage precipitation of raster *x*; *AET_xj_*:evapotranspiration of raster *x* in land use type *j.* |
| Soil Erosion | modified universal soil lossequation (RUSLE) | A = R*K*LS*C*P  A: the amount of soil conservation; R: thefactor of rainfall erosivity; K:factor ofsoil erodibility; L and S: are thetopographic factors. |
| Carbon Sequestration | InVEST carbon storage and  sequestration model | Ctotal = Cabove + Cbelow + Csoil + Cdead  Ctotal: the total amount of carbon storage; Cabove: above-ground biomass carbon stocks; Cbelow: below-ground biomass carbon stocks; Csoil: soil carbonstocks; Cdead: dead organic matter carbon stocks. The biomass carbon density for the different land-use types was derived from the results of a relevant study. |

Table S3 Landscape Fragmentation Index of Northeast China in Current and Future

| **Scenario combination** | **NP** | **PD** | **AREA_MN** | **SHAPE_AM** | **FRAC_AM** | **DIVISION** | **AI** |
| --- | --- | --- | --- | --- | --- | --- | --- |
| Current | 82024.000 | 0.067 | 1503.000 | 43.975 | 1.255 | 0.922 | 69.347 |
| 2030s-SSP126 | 20099.000 | 0.016 | 6133.743 | 25.920 | 1.233 | 0.854 | 88.687 |
| 2030s-SSP245 | 19831.000 | 0.016 | 6216.636 | 27.408 | 1.237 | 0.848 | 88.447 |
| 2030s-SSP585 | 18695.000 | 0.015 | 6594.389 | 26.552 | 1.236 | 0.841 | 88.872 |
| 2050s-SSP126 | 15627.000 | 0.013 | 7889.045 | 24.083 | 1.230 | 0.843 | 90.289 |
| 2050s-SSP245 | 17462.000 | 0.014 | 7065.096 | 26.063 | 1.235 | 0.840 | 89.305 |
| 2050s-SSP585 | 16970.000 | 0.014 | 7269.929 | 24.553 | 1.230 | 0.837 | 89.613 |

Table S4 Current and Future Soil Erosion Values of Northeast China and Each Province

| **Soil erosion (t*km^-2^*a^-1^)** | **Current** | **2030s** | | | **2050s** | | |
| --- | --- | --- | --- | --- | --- | --- | --- |
|  |  | **SSP126** | **SSP245** | **SSP585** | **SSP126** | **SSP245** | **SSP585** |
| Northeast China | 719.214 | 933.782 | 874.935 | 1045.851 | 973.471 | 871.001 | 1131.946 |
| Heilongjiang Province | 482.781 | 520.179 | 594.692 | 497.757 | 504.402 | 503.815 | 551.415 |
| Jilin Province | 835.738 | 1143.964 | 1283.964 | 1192.916 | 1110.979 | 1476.714 | 1283.292 |
| Liaoning Province | 1126.919 | 1292.719 | 1408.479 | 1139.756 | 1221.984 | 1482.040 | 1589.117 |
| Inner Mongolia Autonomous Region | 775.612 | 1140.597 | 1276.871 | 1038.617 | 1032.387 | 1066.633 | 1496.378 |


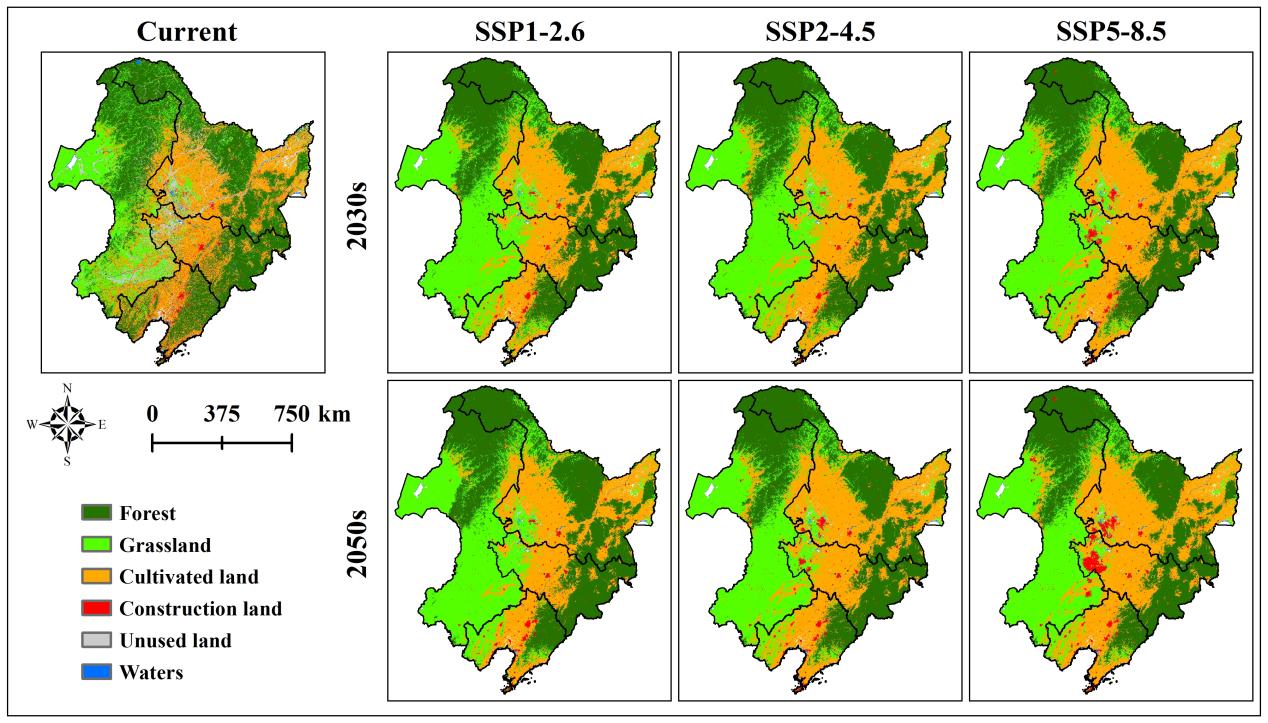


Figure S1 Current and Future Land Use in Northeast China

Table S5 Current and Future Habitat Quality Values of Northeast China and Each Province

| **Habitat quality** | **Current** | **2030s** | | | **2050s** | | |
| --- | --- | --- | --- | --- | --- | --- | --- |
|  |  | **SSP126** | **SSP245** | **SSP585** | **SSP126** | **SSP245** | **SSP585** |
| Northeast China | 0.635 | 0.680 | 0.689 | 0.672 | 0.664 | 0.656 | 0.642 |
| Heilongjiang Province | 0.629 | 0.662 | 0.657 | 0.636 | 0.666 | 0.644 | 0.621 |
| Jilin Province | 0.584 | 0.647 | 0.644 | 0.614 | 0.663 | 0.631 | 0.584 |
| Liaoning Province | 0.543 | 0.557 | 0.545 | 0.528 | 0.571 | 0.533 | 0.515 |
| Inner Mongolia Autonomous Region | 0.692 | 0.749 | 0.738 | 0.733 | 0.760 | 0.738 | 0.728 |

Table S6 Current and Future Carbon Storage Values of Northeast China and Each Province

| **Carbon storage**  **(t/a)** | **Current** | **2030s** | | | **2050s** | | |
| --- | --- | --- | --- | --- | --- | --- | --- |
|  |  | **SSP126** | **SSP245** | **SSP585** | **SSP126** | **SSP245** | **SSP585** |
| Northeast China | 11872.227 | 12338.803 | 12504.955 | 12079.659 | 11855.421 | 11737.598 | 11561.989 |
| Heilongjiang Province | 11820.365 | 12467.523 | 12241.078 | 11775.083 | 12490.540 | 11878.042 | 11556.448 |
| Jilin Province | 11392.850 | 12206.502 | 11949.161 | 11366.713 | 12451.102 | 11616.546 | 10964.995 |
| Liaoning Province | 11180.673 | 9687.653 | 9330.299 | 8963.553 | 9956.101 | 9033.519 | 8828.742 |
| Inner Mongolia Autonomous Region | 12343.025 | 13104.212 | 12843.084 | 12732.190 | 13346.309 | 12824.316 | 12681.112 |

Table S7 Current and Future Water Retention of Northeast China and Each Province

| **Water retention**  **(mm)** | **Current** | **2030s** | | | **2050s** | | |
| --- | --- | --- | --- | --- | --- | --- | --- |
|  |  | **SSP126** | **SSP245** | **SSP585** | **SSP126** | **SSP245** | **SSP585** |
| Northeast China | 8.196 | 11.905 | 12.144 | 12.707 | 13.996 | 12.442 | 14.369 |
| Heilongjiang Province | 11.423 | 13.033 | 14.579 | 13.831 | 13.590 | 14.364 | 14.531 |
| Jilin Province | 8.349 | 16.190 | 16.796 | 18.289 | 16.890 | 20.497 | 20.924 |
| Liaoning Province | 13.376 | 21.945 | 21.570 | 21.791 | 22.191 | 27.552 | 27.122 |
| Inner Mongolia Autonomous Region | 3.307 | 5.821 | 6.343 | 5.662 | 5.551 | 6.622 | 7.429 |

Table S8 Comprehensive Resistance Values of Each Province in Northeast China under Different Carbon Emission Scenarios

| **Mean value** | **SSP126** | **SSP245** | **SSP585** |
| --- | --- | --- | --- |
| Heilongjiang Province | 0.072 | 0.071 | 0.072 |
| Jilin Province | 0.087 | 0.088 | 0.088 |
| Liaoning Province | 0.083 | 0.083 | 0.084 |
| Inner Mongolia Autonomous Region | 0.098 | 0.098 | 0.099 |
